# Phytochemicals Involved in Mitigating Silent Toxicity Induced by Heavy Metals

**DOI:** 10.3390/foods13070978

**Published:** 2024-03-22

**Authors:** Jessica Ceramella, Azzurra Chiara De Maio, Giovanna Basile, Anastasia Facente, Elisabetta Scali, Inmaculada Andreu, Maria Stefania Sinicropi, Domenico Iacopetta, Alessia Catalano

**Affiliations:** 1 Department of Pharmacy, Health and Nutritional Sciences, University of Calabria, 87036 Cosenza, Italy; jessica.ceramella@unical.it (J.C.); azzurrademaio@hotmail.it (A.C.D.M.); biologanutrizionistagb@gmail.com (G.B.); anastasiafacente_93@hotmail.it (A.F.); domenico.iacopetta@unical.it (D.I.); 2 Unit of Dermatology, Spoke Hospital, Locri, 89044 Reggio Calabria, Italy; elisabettascali@libero.it; 3 Departamento de Química, Universitat Politècnica de València, Camino de Vera s/n, 46022 Valencia, Spain; 4 Unidad Mixta de Investigación UPV-IIS La Fe, Hospital Universitari i Politècnic La Fe, Avenida de Fernando, Abril Martorell 106, 46026 Valencia, Spain; 5 Department of Pharmacy-Drug Sciences, University of Bari “Aldo Moro”, 70126 Bari, Italy; alessia.catalano@uniba.it

**Keywords:** heavy metals, phytoantioxidants, plant and herbal extracts, probiotics, chelation therapy, human health

## Abstract

Heavy metals (HMs) are natural elements present in the Earth’s crust, characterised by a high atomic mass and a density more than five times higher than water. Despite their origin from natural sources, extensive usage and processing of raw materials and their presence as silent poisons in our daily products and diets have drastically altered their biochemical balance, making them a threat to the environment and human health. Particularly, the food chain polluted with toxic metals represents a crucial route of human exposure. Therefore, the impact of HMs on human health has become a matter of concern because of the severe chronic effects induced by their excessive levels in the human body. Chelation therapy is an approved valid treatment for HM poisoning; however, despite the efficacy demonstrated by chelating agents, various dramatic side effects may occur. Numerous data demonstrate that dietary components and phytoantioxidants play a significant role in preventing or reducing the damage induced by HMs. This review summarises the role of various phytochemicals, plant and herbal extracts or probiotics in promoting human health by mitigating the toxic effects of different HMs.

## 1. Introduction

**Figure 1:**
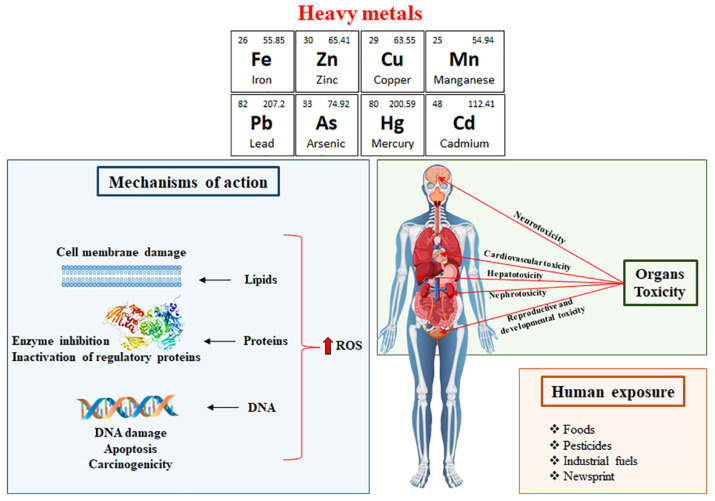
Mechanism and organ toxicity following human exposure to HMs.

Silent toxicity induced by HMs in the environment is a concerning issue that can have serious health implications for consumers. Nutraceuticals are dietary supplements or food products that are demonstrated to provide health benefits beyond essential nutrition and include vitamins, minerals, herbal extracts, other bioactive compounds and so on. However, contamination with HMs such as lead, cadmium, mercury and metalloids, such as arsenic can occur during the production or sourcing of nutraceutical ingredients, leading to potential health risks (Figure 1) [1]. In this context, in the past few decades, the significance of environmental pollutants in impacting human health has become increasingly evident, making them recognised as a crucial public health concern [2]. HMs are natural elements present in the Earth’s crust that play a pivotal role as environmental contaminants. Once released, they endure in the environment for extended periods because of their non-degradable nature, and may potentially induce toxicity in microorganisms, plants, animals and humans [3]. Arsenic (As) is considered to be part of the HMs group because of its high atomic weight and density [4,5] (5.73 g/cm^3^). At low concentrations, several HMs, including iron (Fe), zinc (Zn), copper (Cu) and manganese (Mn), are essential for human survival but can become toxic agents at higher concentrations. In contrast, other HMs, such as mercury (Hg), As, lead (Pb) and cadmium (Cd), have no biological role [6,7,8,9] and enter the human body due to their presence in the environment [10] or as silent poisons in our daily products and diets (foods, pesticides, industrial fuels and even in the newsprint), leading to several serious health consequences [11,12]. In the last few decades, great attention has been paid to the health risks of HMs in road dust [13,14]. Roy et al. (2022) [15] reviewed the state of contamination from HMs, specifically the ecotoxicological and human health risks of HMs described in urban road dust from various cities in diverse continents (Europe, Africa, Asia, America and Australia). The concentrations of HMs were higher than their background values in soil, and the levels of contamination differed depending on the cities, the countries, the continents and the periods under study. Hg, Pb, Cd and As are the most common HMs able to induce human poisonings. In particular, Hg is a ubiquitous metal existing in three different forms (elemental, inorganic and organic) that induces toxic effects, showing different toxicological profiles. The main mechanisms related to Hg toxicity are represented by its interaction with the sulfhydryl groups, the induction of oxidative stress and the disruption of calcium ion homeostasis [16]. The exposure to Pb also represents a major public health risk, since it generates several dramatic effects on the haematopoietic, renal, reproductive and central nervous system, principally through enhanced oxidative stress [17]. Cd affects cell proliferation, differentiation and apoptosis, interfering with DNA repair mechanisms and reactive oxygen species (ROS) generation [18]. Finally, exposure to As, present in various diverse chemical forms and oxidation states, produces acute and chronic adverse health effects. The high toxicity of this metal depends on its metabolism in the trivalent or pentavalent state. In the trivalent state, As can react with essential thiols in proteins, inhibiting their activity. Moreover, other potential mechanisms comprise genotoxicity, altered DNA methylation, oxidative stress and tumour promotion [19].

The health risks associated with HMs can occur either indirectly, through the consumption of vegetables, fruits or cereals grown in contaminated soils, or directly through the inhalation of dust or the consumption of contaminated drinking water. When the concentrations of HMs exceed a certain threshold, they induce toxic effects [20,21]. Regulatory agencies in several countries have determined the maximum allowable limits for HMs in dietary supplements and nutraceuticals to protect consumers. These limits may vary, depending on the type of product and the specific HMs. The World Health Organisation (WHO) established the acceptable limits (minimum and maximum) for drinking water and their adverse effects. For instance, the reported values indicate that the maximum acceptable limit in drinking water is 0.05 ppm for Pb, As and Cr, while it becomes smaller for Cr (0.02) and, especially, for Hg (0.001) [22]. Concerning foods, an in-depth study of the literature has shown that Hg/MeHg was the most studied HM in fish and shellfish foods, in which the FAO/WHO maximum limit is around 1.2 and 1.6 ppm, depending on the fish type. In the cereals group, Cd and As levels in rice were the most reported, with a maximum limit of 0.4 and 0.2 ppm, respectively. Finally, in vegetables, the leafy and fruiting vegetables were the most reported, in which the Cd maximum limits are 0.2 and 0.05 ppm and the Pb maximum levels are 0.3 and 0.05 ppm, respectively [23].

In this context, HM toxicity represents a complex phenomenon attributable to their pleiotropic effects, which disrupt numerous metabolic processes and lead to ultrastructural modifications in exposed cells [24]. The toxic action exerted by HM ions is related to four different mechanisms: (1) binding to thiol, carboxyl and histidyl groups of proteins and glutathione (GSH), thus leading to the impairment of activity, structure disruptions and alterations in regulation and signalling pathways; (2) replacement of essential metal ions present in several enzymes, which may determine impairment of the activity of these enzymes; (3) analogy to biochemical functional groups, especially phosphate; and (4) production of ROS through auto-oxidation [25]. Thus, the exposure of the human biological system to HMs initiates oxidative stress, which further disrupts protein functioning and results in damage to DNA and lipid peroxidation [26,27,28]. The approach used to remove HMs involves the use of chelating agents, which are compounds able to interact with metal ion-forming structures, named chelates [29]. Several literature studies and reviews report that the administration of dietary supplements rich in antioxidant compounds along with chelating agents has improved their therapeutic efficacy. Furthermore, the use of foods, dietary supplements and natural phytoantioxidants can serve as an alternative to the limitations associated with traditional metal chelators (numerous side effects due to the loss of essential metals from the body) [30,31]. In the last decade, dietary components have shown the ability to reduce the toxicity of environmental pollutants, and a rising body of scientific evidence indicates that phytoantioxidants, particularly flavonoids, play a part in preventing or reducing the damage caused by HMs, owing to their antioxidant and metal-chelating properties [2]. Phytochemicals were shown to mitigate HM-mediated toxicity [32], specifically from As [33,34], arsenic trioxide [35], Cd and Pb [36]. Many flavonoids, such as gossypin (3,5,8,3′,4′-pentahydroxy-7-*O*-glucosyl flavone, originally isolated from *Hibiscus vitifolius*) and quercetin have recently gained attention for their potential role in reducing the accumulation of HMs in biological systems [30]. Furthermore, several plants, including *Hippophae rhamnoides*, *Centella asiatica*, *Aloe vera* and others, along with their extracts, are investigated for their significant role in mitigating the toxic effects caused by HMs [7]. Given this context, the aim of this narrative review is to outline how various phytochemicals, through diverse mechanisms, contribute to promoting health and preventing diseases by mitigating the toxic effects of HMs.

## 2. Phytochemicals Used to Ameliorate Heavy Metal-Induced Toxicity

To date, chelation therapy has been the most important treatment for HM toxicity [37]. However, these chelating agents are related to specific drawbacks and undesirable side effects [38]. The most frequent disadvantages of metal chelators comprise the redistribution of some HMs from different tissues to the brain, thus causing neurotoxicity. In addition, HMs may react with essential metals, including copper and zinc, leading to possible serious side effects, such as hepatotoxicity [39]. The use of natural agents was proposed for the prevention of HM toxicity. Natural supplements, herbs, foods and phytoantioxidants can alleviate metal poisoning, thanks to their affordability and minimal side effects. The toxicity of HMs is primarily attributed to oxidative stress [40], which can be defined as a disproportion between the production of free radicals and reactive metabolites, the oxidants and their removal by antioxidant systems [41]. Nevertheless, the antioxidant mechanism is not the only one responsible against toxicity, since binding may also be beneficial. The efficacy of treatment with antioxidants is strongly associated with chelation therapy for the treatment of several diseases [42]. Furthermore, antioxidants are not clinically adopted against heavy metal-induced cell damage, even though some recent outcomes suggested their protective effect [28].

**Figure 2:**
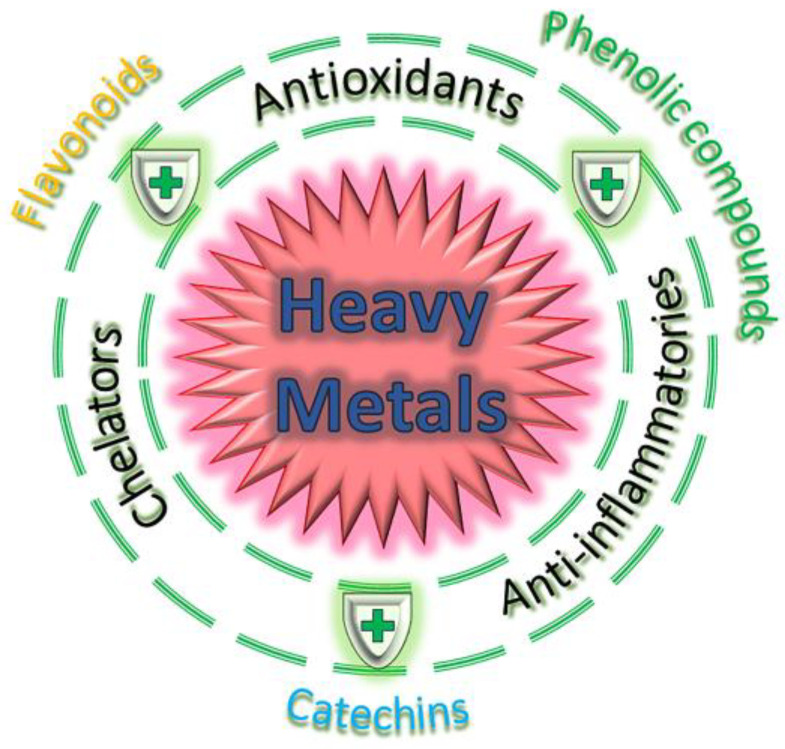
An overview about the protective role of the most representative phytochemicals from HM toxicity.

Many edible plants and foods are known for their antioxidant behaviour since they are abundant in phytoantioxidants [43] and act as preventive measures against HM toxicity [44]. These natural antioxidants encompass flavonoids, phenolic compounds, isoflavones, lutein, lycopene, carotenoids and tocopherols, which may inhibit oxidation, act as free radicals scavengers and function as chelators and reductants (Figure 2).

Many spices, herbs, fruits and vegetables curb high levels of phenols [29]. Chemically, these compounds bear one or more aromatic rings with one or more phenolic groups able to donate protons to the oxidising or pro-oxidative compound [45]. Indeed, the mechanism by which the HMs induce toxicity mainly follows the free radical production pathway(s), especially the generation of ROS and reactive nitrogen species (RNS). The over-production of these free radicals creates a discrepancy between the oxidative and antioxidative systems, which may lead to oxidative stress emergence, which may cause necrosis, DNA damage and many chronic and degenerative disorders. It is known that the phytochemicals containing flavonoids and polyphenols may ameliorate HM-mediated toxicity in humans [46,47,48].

In particular, the antioxidant properties of phenolic acids mainly rely on the number of hydroxyl groups and on the steric effects they may exert, depending on the position of the hydroxyl groups, as well as the type of substitution on the aromatic ring [49].

Regarding the antioxidant properties of flavonoids, they are related to the presence of the catechol group, which displays a significant ability to “scavenge” ROS and RNS and ensures the high stability of the created phenoxyl radical (hydroxyl groups are donors of electrons and nitrogen for radicals), has a double bond between the C-2 and the C-3 and the presence of the 4-oxo group and hydroxyl groups near C-3 and C-5 in the presence of 4-oxo groups, which generate the maximum free radical scavenging effect [50].

Many flavonoids may chelate transition metal ions. This is due to the ability of the many hydroxyl groups to form complexes with metals, preventing their gastrointestinal absorption and accelerating their elimination through urine [51]. Polyphenols are also capable of inhibiting the activity of enzymes that participate to the formation of reactive forms of oxygen.

For example, the -OH phenolic group of quercetin binds essential amino acid residues in the active sites of the enzymes acetylcholinesterase and butyrylcholinesterase, inhibiting their oxidative activities [52]. Moreover, Odbayar et al. (2009) found that quercetin enhances the effect of antioxidant enzymes, such as GSH transferase and aldo-keto reductase [53].

Curcumin’s antioxidant effect is principally related to its chemical structure, as it bears phenolic and methoxy groups on the phenyl ring and 1,3-diketone moiety, and to its ability to remove hydroxyl radicals, singlet oxygen and RNS [54]. 

Regarding lycopene, carotenoids and tocopherols, the effects are probably due to their free-radical scavenging and antioxidant functions. They are probably involved in the scavenging of two ROS, the singlet molecular oxygen (^1^O_2_) and the peroxyl radicals. The interaction of carotenoids with ^1^O_2_ is largely related to physical quenching, which implicates a direct energy transfer between the two molecules. The effectiveness of carotenoids for physical quenching depends on the number of conjugated double bonds [55].

It is also known that phytochemicals, taken together with other chemotherapeutic agents, may be useful for the management of toxicity induced by HMs. The co-administration of phytochemicals with chelating agents was demonstrated for As, leading to a more pronounced elimination of As from the body, with lesser off-site adverse effects. The combination treatment of the chelating agent with a phytochemical allows for the use of a lower dose of the chelating agent, without compromising the treatment [33]. However, although the co-administration of natural antioxidants with known chelating agents was shown to ameliorate the removal of toxic metals, extensive clinical studies to determine the appropriate dose and treatment duration are still needed [56].

### 2.1. Flavonoids

**Figure 3:**
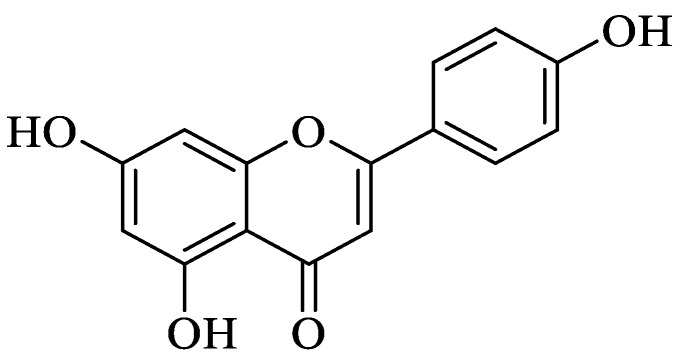
Chemical structure of flavonoids.

Flavonoids (Figure 3) are secondary metabolites commonly found throughout the plant kingdom and abound in plant-derived foods and beverages, including fruits, vegetables, tea, cocoa and wine.

They play a part in the aroma and colour of fruits and flowers, perform to protect plants from diverse biotic and abiotic stress, including UV radiation, frost and droughts and possess antibiotic properties [57]. The antioxidant action of flavonoids has been remarkably emphasised in recent years. This activity is related to free radical scavenging, chelation of transition metal ions, increase in GSH content and modulation of defensive gene expression via the nuclear erythroid 2-related factor 2/antioxidant response element (Nrf2/ARE) pathway, which is an inherent defensive mechanism towards oxidative stress. Moreover, flavonoids, apart from their activity as free radical scavengers [58] and regulators of defensive gene expression, are also excellent chelators [59]. The free radical scavenging characteristics of flavonoids may be attributed to the amount and position of the free −OH groups in their molecular structure [60]. Flavonoids with numerous hydroxyl groups exhibit higher antioxidant potency than those bearing only one hydroxyl group. Moreover, the presence of the *ortho*-3,4-dihydroxy moiety improves the antioxidative action [61]. Furthermore, they can reduce the oxidation promoted by transition metals, potentially by donating them a hydrogen atom, leading to a lower pro-oxidative action. Flavones and specific flavanones, including naringenin, may selectively bind metals through their 5-hydroxyl and 4-oxo groups [61]. Among the multiple flavonoids, gossypin and quercetin have recently attracted attention for their potential to mitigate the accumulation of HMs within internal biological systems. Recent research by Li et al. (2017) [62] revealed that flavonoids have cytoprotective effects by ameliorating the activity of antioxidation enzymes, countering Cd-induced toxicity. Particularly, the cytoprotective effect can be principally attributed to three mechanisms. Firstly, flavonoids help clear ROS, thus reducing the production of lipid peroxides and enhancing the antioxidant enzyme activity. Secondly, flavonoids chelate Cd, thus lowering its accumulation and modifying the levels of other essential metal ions in vivo. Lastly, these compounds may contribute to the reduction of DNA damage and the inhibition of apoptosis.

#### 2.1.1. Quercetin

Quercetin (3′,3,4′,5,7-pentahydroxyflavone, Table 1), a flavonoid found in fruits, vegetables, olive oil, red wine and tea, was observed to neutralise free radicals induced by HM exposure [63,64]. Quercetin may be used alone or in association with a chelating agent for As exposure [56]. Mishra and Flora (2008) [65] studied the effectiveness of quercetin and monoisoamyl 2,3-dimercaptosuccinic acid (MiADMSA), alone or in association, towards arsenic-induced oxidative stress and the mobilisation of As in mice. Animals were chronically exposed to 25 ppm sodium arsenite in drinking water for 12 months followed by treatment with oral MiADMSA (0.2 mmol/kg) and quercetin (0.2 mmol), alone or in combination, once daily for 5 consecutive days. The exposure to As led to a substantial depletion of blood δ-aminolevulinic acid dehydratase action, GSH, white blood cell and red blood cell counts, and an increase in platelet levels together with a significant increase in ROS levels (red blood cells). Quercetin administration induced a depletion in As levels when compared with the administration of MiADMSA only from target organs, including the blood (3.12 ± 0.21 vs. 5.34 ± 0. 35 ng/dL), the liver (3.14 ± 0.15 vs. 4.06 ± 0.16 µg/g of tissue) and the kidneys (1.68 ± 0.29 vs. 1.92 ± 0.23 µg/g of tissue). Moreover, it was evidenced that a nanocapsulated drug delivery system for quercetin may provide more efficient protection against As-induced damage compared to the bulk administration of quercetin [66]. Indeed, nanocapsulated quercetin-treated rats showed 4-fold lower levels of As concentration in liver and brain mitochondria when compared with the sodium arsenite-treated rats. In addition, it is possible to observe a statistically significant difference even with free quercetin-treated rats. Cai et al. (2021) [67] studied the effect of quercetin and allicin against Pb poisoning in chickens, which improved the body’s antioxidant defence. The authors demonstrated that quercetin and allicin could ameliorate oxidative damage and apoptosis in the Pb-poisoned chickens and alleviate liver tissue damage caused by Pb through the PI3K signaling pathway, with stronger effects achieved with their combination. Recently, Srivastava et al. (2023) [68] studied the potential of quercetin to protect Cd-induced cognitive deficits in rats, obtained by the modulation of NMDA-R-mediated downstream signaling and the PI3K/AKT—Nrf2/ARE signaling pathways in the hippocampus. The treatment with quercetin (25 mg/kg b.w. p.o.) for 28 days attenuated Cd-induced changes in the hippocampus. A recent study by Al-Zharani et al. (2023) [69] reported the effect of quercetin in reducing the oxidative stress induced by Pb toxicity in male Wistar rats. Quercetin was given at 350 mg/kg b.w. for two months. The authors found that the levels of haematological values in rats treated with quercetin were brought back to control levels, thus suggesting the capacity of quercetin to remarkably diminish the negative effects of Pb on the blood cells. A direct chelating action or, in the alternative, a blockade of the Pb-mediated reactions that damage the circulating blood cells were likely involved.

#### 2.1.2. Hesperidin and Hesperetin

Hesperidin and hesperetin are flavonoids found in citrus fruits which may counteract the molecular alterations and toxic effects induced by HMs. They reduce HM toxicity through anti-inflammatory, antioxidant and anti-apoptotic activities, obtained by scavenging free radicals and modulating ATPases, cell cycle proteins and diverse cellular signalling pathways [70]. The catechins present in green tea have displayed comparable protective effects in instances of Pb and Cd poisoning [56]. Khuntia et al. (2023) [71] reported the ability of hesperidin (100 mg/kg b.w. p.o.) to attenuate cardiac toxicity from arsenic trioxide in rats. Cardiac toxicity was triggered by the administration of arsenic trioxide (4 mg/kg, orally) for one month. The authors found that arsenic trioxide-induced histopathological injury to cardiac tissue was clearly alleviated with hesperidin treatment. This was evidenced by reduced creatine kinase–myoglobin binding and lactate dehydrogenase levels, improving heart rate and alleviating histopathological damage to the heart. Abu-Khudir et al. (2023) [72] studied the effect of hesperidin on Pb-induced reprotoxicity, a negative consequence of Pb exposure, which leads to anomalous spermatogenesis, testicular degeneration and pathogenic sperm modifications in Wistar rats. The treatment with hesperidin (100 mg/kg b.w.) markedly restored the Pb acetate-induced reduction in body, epididymal and testicular weights, as well as semen parameters, reproductive hormones and testicular markers of oxidative stress. Decreased malondialdehyde levels and enhanced testicular histopathological findings were also observed. Thus, hesperidin was suggested for the prevention of testicular injury induced by Pb, mediated through the suppression of oxidative stress.

Bernhoft et al. (2013) [73] studied the effects of hesperetin against Cd-induced neurodegeneration and memory damage in rat brains. Hesperetin was administered at 40 mg/kg doses for 3 weeks and determined a reduction of oxidative stress, the restoration of mitochondrial dysfunction, reduced apoptosis and up-regulated antioxidant transcription factors. The chronic administration of hesperetin at 10 and 50 mg/kg doses for 5 weeks determined a neuroprotective effect against oxidative stress, as revealed by the reduction of lipid peroxidation and the triggering of the catalases (CATs), total superoxide dismutase (SOD) and GSH-related enzymes. In addition, even at the higher dose, hesperetin did not determine apoptosis in the brain.

### 2.2. Epigallocatechin Gallate (EGCG)

Over the past few years, several studies examined the importance of epigallocatechin gallate (EGCG), a chiral compound [74] that represents the main catechin that makes up 50–80% of the total catechin content in green tea [75] on HM-induced toxicity in both in vitro and in vivo experimental conditions. In general, the in vivo studies indicated that EGCG is able to mitigate HM toxicity through its ability to scavenge ROS, enhance HM excretion, induce the expression of Nrf, exert anti-inflammatory effects and protect mitochondria [76]. The protective effect of EGCG was observed in numerous organs and tissues, such as the liver, testes, kidney, and neural tissue. Despite the limited bioavailability, pharmacokinetic data demonstrate that EGCG is present in various internal organs and tissues, crossing the blood–brain barrier to reach tissues impaired by HM poisoning. Nevertheless, the results from in vitro models are inconsistent. Several studies, especially those using cell culture models, indicated that EGCG can exert opposing effects and potentiate the harmful consequences of HM exposure. This variability could be due to differences in the susceptibility of specific cell types or variations in cell culture conditions, such as the inadvertent generation of hydrogen peroxides or other reactive molecules due to the auto-oxidation of EGCG in cell culture media [76]. A study by Iheanacho et al. (2023) [77] investigated the potential effect of EGCG in attenuating As-induced acute injury and long-term exposure-associated fibrogenecity in vitro in two human kidney epithelial cell lines, Caki-1 and HK-2. The latter were exposed to As, for both acute and long-term durations, then treated with EGCG. Results showed that EGCG has a protective effect in As-induced acute cytotoxicity in these cells. Another study carried out in vitro (H9c2 cells) and in vivo (rats) suggested that EGCG is able to attenuate sodium arsenite (NaAsO_2_)-induced cardiac injuries, oxidative stress, intracellular calcium accumulation and apoptosis [78]. Parasuraman et al. (2020) [79] studied the effect of EGCG in combination with vitamin C on cadmium chloride (CdCl_2_)-induced oxidative stress in female Sprague Dawley rats. The coadministration of EGCG with vitamin C was shown to prevent the CdCl_2_-induced oxidative stress. The CdCl_2_-administered group showed a significant increase in the levels of glucose, AST, ALT and urea when compared with the control group, whereas vitamin C and EGCG prevented the CdCl_2_-induced biochemical changes. Vitamin C and EGCG also prevented the CdCl_2_-induced reduction in the levels of GSH and CAT. An et al. (2014) [80] studied the effects of EGCG in Cd^2+^-induced apoptosis in normal human liver cells (HL-7702) to clarify the effect and detailed mechanism of EGCG. Cd^2+^ significantly reduced the cell viability and induced apoptosis. Conversely, EGCG co-treatment determined a high inhibition of Cd^2+^-induced reduction of cell viability and apoptosis, thus implying a rescue effect of EGCG against Cd poisoning. The authors concluded that the protective effect most likely arises from the scavenging ROS activity and maintenance of redox homeostasis, rather than the metal-chelating properties.

### 2.3. Curcumin

Curcumin is a polyphenolic compound naturally found in high concentrations in the rhizome of the plant *Curcuma longa*, generally known as turmeric [81]. The reactivity of its α-β-unsaturated β-diketone moiety gives it a good chelating activity for metal ions as it strongly coordinates many metal ions including Cu^2+^, Zn^2+^, Pb^2+^ and Cd^2+^ [82,83]. In addition, curcumin is able to induce detoxifying enzymes by up-regulating the Kelch-like ECH-associated protein 1 (Keap1)/Nrf2/ARE pathway and it is able to down-regulate NF-kB and the expression and concentration of proinflammatory cytokines by preventing the damaging effects in the liver caused by HMs [84]. Curcumin’s effects against HM-induced toxicity were investigated in several studies. The role of curcumin in Hg toxicity was examined by Agarwal et al. (2010) [85] by administering curcumin both before and after Hg exposure to evaluate its preventive and therapeutic effects. They found that daily treatment with curcumin (80 mg kg^−1^ b.w. for 3 days, orally) was effective in preventing Hg toxicity, as evidenced by reduced levels of lipid peroxidation (LPO), reduced GSH and creatinine levels, and alterations in endogenous enzyme activities observed in liver, kidney, brain, and blood tissues. Moreover, the groups treated with curcumin before and after Hg exposure also exhibited lower concentrations of Hg in the liver, kidneys, and blood compared to the corresponding groups treated with Hg alone. In particular, the post-treatment with curcumin significantly reduced the accumulation of Hg by about 3-fold in these tissues, mostly in the liver and kidneys. Thus, curcumin was suggested as a therapeutic agent after Hg intoxication. Abdel-Moneim et al. (2015) [86] investigated the protective effects of curcumin against haematological and biochemical alterations, along with renal pathologies induced by Pb in male albino rats. They were intraperitoneally exposed to lead acetate (25 mg/kg b.w., once a day) either alone or in combination with curcumin (30 mg of curcumin/kg b.w., twice a day) for 7 days. The exposure of rats to Pb^2+^ resulted in considerable reductions in haemoglobin levels, haematocrit values and platelet counts, as well as a remarkable increase in LPO and a substantial reduction in total antioxidant capacity. The co-administration of curcumin to the Pb-treated rats restored a major part of the above-mentioned parameters to levels that were almost normal. Moreover, a 2- or 3-fold significant decrease in Pb^2+^ concentration was observed in the Pb^2+^ plus curcumin group compared to that in the Pb^2+^-alone-treated group. Recently, Rahaman et al. (2020) [87] evidenced that pre-treatment with curcumin significantly improved As-induced cytotoxicity by leveraging its antioxidant properties. As exerted cytotoxic effects on pheochromocytoma (PC12) cells, inducing both autophagic and apoptotic cell death. Curcumin demonstrated an antioxidant effect by modulating the Nrf2 antioxidant signalling pathway and reduced the toxicity triggered by As in PC12 cells with the regulation of autophagy and apoptosis. Additionally, curcumin functioned as an effective metal-chelating agent. The resultant curcumin–metal complexes boosted the antioxidant properties of curcumin. Thereby, curcumin utilisation in chelation therapy was suggested for the prevention of metal-induced neurodegenerative diseases, such as Alzheimer’s disease [88].

### 2.4. Ferulic Acid

Ferulic acid (FA, [*E*]-3-[4-hydroxy-3-methoxy-phenyl]prop-2-enoic acid) is an organic compound that belongs to the phenolic acids [89]. FA is most frequently found in whole grains, spinach, parsley, grapes, rhubarb and cereal seeds, especially in wheat, oats, rye and barley [90,91]. It exhibits low toxicity and serves numerous physiological functions [92]. Several studies have suggested that FA may be utilised to mitigate HM toxicity. Its capability to inhibit HM toxicity is strictly related to its antioxidant properties [93]. Kassab et al. (2020) [94] evaluated the protective effect of FA on Cd-mediated reproductive toxicity in male rats, demonstrating that FA supplementation could abolish the testicular damages coupled with metal exposure. Indeed, FA pre-treatment effectively prevented the accumulation of Cd in testicular tissue, reducing Cd concentration by about 2-fold, when compared with the CdCl_2_-alone-treated rats. Previously, Sanjeev et al. (2019) [95] suggested that FA supplementation might significantly prevent oxidative stress and restore histological parameters of the liver and kidneys of rats, thus indicating its hepato- and nephron-protective, antioxidant and anti-inflammatory effects. Moreover, the concentration of Cd was found to be higher in the liver than in the kidneys after 15 and 30 days of Cd exposure, and it was significantly reduced by about 5-fold in the rat group co-treated with Cd + FA compared to the Cd-alone-treated group. FA was also suggested as a promising candidate for the prevention of Pb toxicity. Kelainy et al. (2019) [96] demonstrated the protective action of FA against oxidative stress and DNA damage caused by Pb in rats. Pb concentration was considerably increased in the kidneys and testes for rats that received lead acetate (168.51 ± 4.29 and 140.18 ± 8.21 µg/g of dry tissue, respectively). On the other hand, Pb levels were 2-fold lower in the rat group that received FA after Pb and 5-fold decreased in the rat group pre-treated with FA in both organs. Moreover, Yu et al. (2021) [97] proved that FA could prevent Pb-induced cognitive impairments in mice via activation of the Nrf2-mediated antioxidant defence system. Indeed, maternal administration of FA prevents cognitive impairment induced by Pb in mouse offspring. These findings confirm the hypothesis that FA targets Nrf2 and performs a substantial brain-protective effect against Pb-induced neurotoxicity.

### 2.5. Ellagic Acid

**Figure 4:**
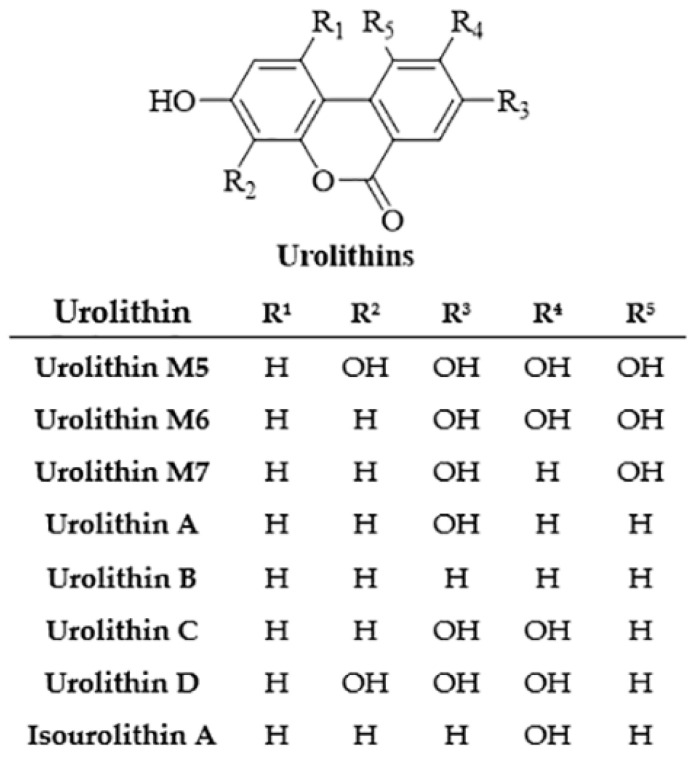
General structure of urolithins, ellagic acid metabolites obtained by the gut microbiota activity.

Ellagic acid (EA) is a natural phenolic compound present in grapes, nuts, strawberries, black currents, raspberries, green tea, pomegranates and the stem and bark of *Eucalyptus globulus*, *Eucalyptus maculata*, and nuts [98]. It is found in its free form or as part of more complex molecules, namely ellagitannins [99]. In the digestion process, ellagitannins form EA, which is, in turn, metabolised by the gut microbiota to diverse dibenzo [*b,d*]pyran-6-one derivatives, called urolithins (Figure 4). The molecule of ellagic acid undergoes cleavage and decarboxylation reactions of one lactone ring, followed by further metabolic dihydroxylation reactions [100].

Recent studies have enhanced the interest in EA as a possible protective agent against kidney toxicity induced by exposure to environmental pollutants. The chelating, antioxidant, anti-inflammatory, anti-autophagic and antiapoptotic properties of EA mitigate HM-induced nephrotoxicity, thus it may prevent the progression of kidney disease [101]. Recently, Iflazoglu Mutlu et al. (2021) [100] investigated the potential protective effects of EA in laying quails exposed to Pb. EA supplementation at a dose of 500 mg/kg enhanced the performance parameters, improved the antioxidant defence system and reduced apoptosis via regulating the expression of caspase-3 and -9. Therefore, the authors concluded that EA could ameliorate the toxic effects related to Pb exposure; however, further studies on this topic are needed.

**Table 1:** Phytochemicals used to ameliorate heavy metal-induced toxicity.

Compd	Name	Ref.
** 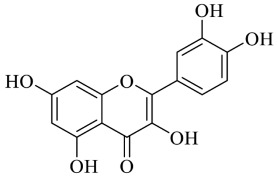 **	Quercetin	[56,65,66]
** 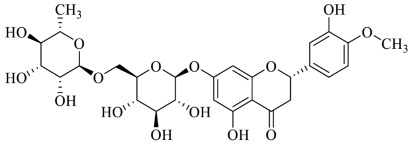 **	Hesperidin	[56,70]
** 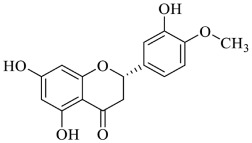 **	Hesperetin	[56,70]
** 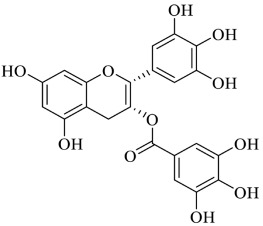 **	Epigallocatechin gallate (EGCG)	[76]
** 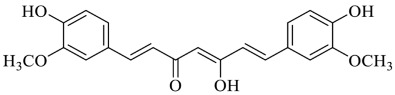 **	Curcumin	[82,83,84,85,86,87,88]
** 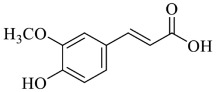 **	Ferulic acid (FA)	[93,94,95,96,97]
** 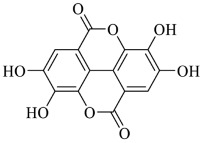 **	Ellagic acid	[100,101]

## 3. Plant and Herbal Extracts in the Management of Heavy Metal-Induced Toxicity

Numerous plants and extracts are studied for their important role in the decrease of toxic effects caused by HMs. Several studies demonstrated that garlic (*Allium sativum* L.), *Centella asiatica* and *Aloe vera* (*Aloe barbadensis* L.) extracts may help to reduce hepatic and renal HM-mediated toxicity in experimental animals. Plant extracts contain numerous phytochemical species that may form complexes with HM, thus protecting cells from oxidative stress [32]. In addition, interesting properties in reducing HM-mediated toxicity were demonstrated by the alga *Spirulina* [102] and the powder of Ginger (*Zingiber officinale*).

Particularly, Mumats et al. (2020) [103] reported the therapeutic role of *A. sativum* L. extract against Pb toxicity in various organs. The pre-treatment with garlic extracts along with antioxidants, like vitamins C and E, decreased oxidative stress, restoring the biochemical alterations in the testes, liver, brain, kidneys and bone. Reddy et al. (2012) [104] reported that supplementation of Pb acetate for 20 and 40 days at a dose of 75 mg/kg b.w. determined a mild hyperplasia, whereas supplementation of garlic juice and vitamin C in pregnant mice exposed to Pb acetate prevented malformation of the bones of mice neonates. Pourjafar et al. (2007) [105] treated mice with 5 mg/kg b.w. of Pb acetate and described that it accumulated in bones and other organs. The administration of 125, 250 and 500 mg/kg of garlic extracts reduced the levels of Pb in bones and soft tissues. Moreover, some researchers used *A. sativum* L. extracts to reduce Cd- and Pb-induced mitochondrial damage and apoptosis in tissue culture models, along with decreasing neural, hepatic, renal and haematological damage in rats [32,106]. Garlic was used at a dose of 1 mL/100 g/b.w. [106]. The high antioxidant potential of garlic may likely derive from the presence of allicin [56].

Hernayanti et al. (2021) [107] demonstrated that the administration of *C. asiatica* extract (100–400 mg/kg) for 21 days was able to ameliorate the Cd-induced hepato-inflammation, by decreasing Cd, TNF-α and COx2 levels and increasing GST and GSH ones. Moreover, Flora et al. (2007) [108] suggested that the treatment with an aqueous extract of *C. asiatica* (100–500 mg/kg) for 5 days allowed for the reversal of As-induced oxidative stress in rats, even though it did not possess chelating property since the levels in the blood and soft tissues remained unvaried.

Hussain et al. (2016) [109] studied the effects of a flavonoid-rich aqueous fraction of *A. barbadensis* L. leaf skin on toxicity induced by Cd in albino rabbits. The oral treatment at 200 mg/Kg/day dose for 40 days lowered the toxicity and oxidative stress induced by Cd in the affected tissues and restored the SOD, CAT and vitamin C and E levels in the liver and kidneys, together with a significant reduction in the Cd concentration. Bhattacharjee et al. (2022) [110] examined the protective effects of aloe emodin, an anthraquinone derivative present in *A. barbadensis* L., against Pb-induced hepatotoxicity in rats. After a 28-day period of oral treatment with aloe emodin (100 mg/kg and 200 mg/kg), the levels of the canonical liver biomarkers were reverted in a dose-dependent manner, suggesting the importance of aloe emodin in the diet as a detoxifying agent against chronic exposition to high Pb levels. Finally, the beneficial phytoextracting properties of *A. barbadensis* L. in cleaning up the contaminated soil or water against different metals, such as Cr (III) and (VI), Ni, Cu, Pb and Cd [111,112,113,114] must also be recalled.

### 3.1. Spirulina

Numerous scientific data indicate that *Spirulina* (*Arthrospira*), a photosynthetic filamentous cyanobacterium usually referred to as blue-green algae, offers relief from metal-induced toxicity, including Cd, Hg, Pb and As [102]. This alga grows in temperate waters worldwide and is considered a functional food thanks to the high amount of proteins, vitamins, minerals, healthy fatty acids and other phytonutrients like various active plant colours [115]. It presents exceptional chelating properties for HMs in humans and in soil, water and sludge as well [116]. *Spirulina*’s effectiveness is due to its antioxidant activity, which can be attributed to the presence of phenolic compounds, such as γ-linolenic acid, α-tocopherol, phycobiliproteins, phycocyanin and various other phytochemicals. These compounds could perform their antioxidant activity individually or in combination (synergistic effects) [117]. Particularly, it was reported that phycocyanin, the most abundant phycobiliprotein in *Spirulina*, can bind with HMs, chelating and removing them [118]. Several preclinical studies, listed in Table 2, show the positive effect of *Spirulina* against As, Cd, Pb and Hg experimentally induced toxicity. However, clinical studies describing the protective effects of *Spirulina* against As toxicity in humans are limited [103]. Misbahuddin et al. (2006) [119], in a randomised placebo-controlled study, showed that the human administration of the extract of *Spirulina* plus zinc (250 mg + 2 mg, respectively, twice daily) for 16 weeks in drinking water may be helpful in chronic As poisoning with melanosis and keratosis. Effectively, *Spirulina* improved the symptoms of arsenical palmer keratosis [120]. In a clinical study by Rahman et al. (2006) [121], it was also shown that arsenicosis could be prevented by treatment with *Spirulina*. *Spirulina* could be mixed into fruit or milk drinks or added to recipes for arsenicosis patients to enhance nutritional value, thus providing health and energy. Finally, in another study, the effect of spirulina was evaluated against As-induced toxicities in ducks. In all the animals treated with arsenic trioxide (100 mg/L) plus *Spirulina* (30, 60 and 120 mg/L in drinking water, once a day for 90 days), a significant reduction of all the biochemical parameters (SGPT, SGOT, ALP, LDH and ACP) and of As levels of about 80% in all organs (liver, kidneys, muscles, intestine, heart, femur, brain and faeces) was recorded when compared with the control groups. The highest percentage reduction (82.98%) was observed in the thigh muscle following the highest dose (120 mg/L) of spirulina along with arsenic trioxide [122]. *Spirulina* was also effective against the toxicity induced by Cd, another widespread pollutant that can induce numerous adverse effects in humans and animals. Indeed, the intragastrical administration of *Spirulina* at 62.5, 125, 250 or 500 mg/kg in ICR pregnant mice significantly decreased the frequency of foetuses with exencephaly, micrognathia and skeletal abnormalities and decreased in a dose-dependent manner lipid peroxidation induced by Cd [123]. Moreover, *Spirulina* demonstrated a protective effect with respect to different parameters in Cd-intoxicated male albino rats (Wistar strain). Indeed, the oral administration of *Spirulina* (500 mg/kg b.w./day) for 30 days increased the Cd-induced decrease in copper, zinc, iron, selenium, GSH, SOD, CAT and GSH peroxidase levels when compared to the control groups. The antioxidant and antiperoxidative effects of *Spirulina* were confirmed by histopathological study in liver and kidney sections [124]. Karadeniz et al. (2009) [125] confirmed the protective action of *Spirulina platensis* on Cd-induced oxidative stress and hepatotoxicity in adult female Wistar albino rats. In addition to As and Cd, the toxic effects of Pb remain a main public health threat. Different data assessed the capability of *Spirulina* to prevent the harmful effects of Pb intoxication. Shastri et al. (1999) [126] demonstrated that *Spirulina* administration in Swiss albino mice at a dose of 800 mg/kg b.w., due to its antioxidant activity, induced a significant enhancement in the survival time and reduced Pb-induced toxicity in terms of testes and animal weight and tubular diameter when compared with the Pb-treated group. Moreover, Upasani and co-workers [127] confirmed that *Spirulina* was able to prevent lipid peroxidation and restore levels of SOD, CAT and GSH to normal in the liver, lung, heart and kidneys of Pb-exposed adult albino rats (Wistar strain), preventing Pb deposition in the brain as well. In addition, there was a 2-fold reduction in the Pb levels in the brains of the animals treated with Pb and Spirulina (1500 mg/kg) compared with the control group. However, no significant changes in Pb levels were observed in the liver, lung, heart and kidneys of the two animal groups. The administration of 300 mg/kg of *Spirulina* was also able to diminish the increase in the number of mast cells due to Pb in the cortex and medulla of adult inbred female Wistar albino rat ovaries during the oestrous cycle [128]. Ponce-Canchihuamán et al. (2010) [129] demonstrated that *Spirulina* restored the biochemical parameters of the liver and kidneys in old Wistar male rats, decreasing the lipid levels both in plasma and liver as well as lipid peroxidation in the liver and kidneys. This ability is due to the radical scavenging activity of its components that induced an increase in the antioxidant status indicators (SOD, CAT and GSH). Finally, the addition of *Spirulina* to the diet of Pb-poisoned mother Wistar rats minimised the deposition and toxicity of this metal in neonate’s brains. The administration of *Spirulina* lowered Pb-induced oxidative stress and recovered normal values for the antioxidant enzyme activities, including GSH, CAT and SOD. Moreover, blood Pb levels were measured in the control and intoxicated groups and the obtained data showed that the supplementation of the diet of lead-intoxicated mothers with spirulina significantly lowered the accumulation of lead in the blood circulation of neonates by 50% [130]. The Kumar research group [131] studied the protective effects of *Spirulina* against Hg toxicity. They assessed that the administration of *Spirulina* (800 mg/kg b.w.) to male Swiss albino mice determined a reduction of the Hg-induced testicular toxicity, reducing the activity of acid phosphatase (ACP) and alkaline phosphatases (ALP) in the testes. The same group demonstrated that *Spirulina* treatment of Swiss albino mice prevented Hg-induced alterations in calcium and iron levels, ACP and ALP activity in serum, lipid peroxidation and GSH levels in the blood [132]. Moreover, supplementation with 800 mg/kg of *Spirulina* resulted in decreased lipid peroxidation levels, SGOT and SGPT activity together with an increase in liver GSH levels [133]. Bashandy et al. (2011) showed that treatment with *Spirulina* (300 mg/kg dissolved in water) in albino rats reversed the significant increase in blood hydroperoxide, AST, ALT, ALP, total cholesterol, triglycerides and low-density lipoprotein cholesterol levels induced by Pb. On the other hand, *Spirulina* induced an increase in plasma protein, high-density lipoprotein cholesterol and hepatic GSH when compared to the Pb-exposed group [134]. Finally, oral pre-treatment of male Wistar albino rats with *Spirulina* (300 mg/kg, b.w.) reduced the extent of the observed Hg-mediated toxicity, by reducing lipid peroxidation products, metal accumulation in the testes, histopathological changes of the testes and spermatozoa abnormalities and, at the same time, reverting the Hg-induced inhibition in enzymatic activities of antioxidant biomarkers (SOD, CAT and GPx) [135].

### 3.2. Ginger (Zingiber officinale)

Ginger (*Zingiber officinale*) is a well-known flowering plant whose rhizome, known as ginger root, is usually used in both culinary and traditional medicine. A growing body of evidence has highlighted the diverse biological activities of ginger, such as protection against male infertility, alleviation of nausea and vomiting, management of diabetes and cancer, as well as its anti-inflammatory, analgesic, anti-obesity activities and various other benefits [136,137]. It was also investigated for the detoxification of HMs. Its major chemical constituents comprise gingerols, polyphenols, monoterpenoids, flavonoids and tannins [138]. Xue et al. (2022) [139] conducted a study on the protective effect of ginger powder against Pb-induced male infertility in adult male rats. They found that ginger (250 mg/kg), particularly when combined with zinc, can mitigate reproductive dysfunction by inhibiting apoptosis through the lowering of oxidative damage and inflammation, thus leading to enhanced reproductive performance. Moreover, Motawee et al. (2022) [140] investigated the potential protective effect of ginger root powder against Cd-induced testicular pathology in rats. Their data suggested that ginger (40 mg/kg/day) exerted a protective effect against the deterioration of testicular tissue structure and function induced by Cd. The protection was achieved by enhancing the antioxidant capacity of testicular tissue and steroid production, leading to improvements in sex hormone levels in the bloodstream. Moreover, the antioxidant effect of leaf ethanolic extracts from *Emblica officinalis* (*E. officinalis*) and *Zingiber officinale* (*Z. officinale*) on As- and Pb-induced toxicity was reported in albino rats. Treatment with these extracts showed a protective role by restoring serum biochemical profiles and antioxidant enzyme levels in the kidney and liver tissues of male rats subjected to arsenic-lead-induced toxicity [141]. Thus, ginger is suggested as a potential therapeutic option for preventing HM toxicity.

## 4. Probiotics in the Prevention of Heavy Metal-Induced Toxicity

The modulation of HM levels involving the use of probiotics is an emerging and cost-effective technique. The ability of probiotics to bind HMs, such as Cd, Pb or As, and facilitate their excretion from the body was explored using the bioremediation approach [142,143]. The term probiotics refers to ‘live organisms that, when administered in sufficient quantities, provide a health benefit to the host’. Numerous bacterial species from the genera *Lactobacillus*, *Lactococcus*, *Bacillus*, *Streptococcus*, *Bifidobacterium*, *Pediococcus* and *Propionibacterium* are well-known probiotics [144]. Among these, the most prevalent probiotic bacterial genera with beneficial effects in humans are *Lactobacillus* and *Bifidobacterium* [145]. Probiotics bind HMs through various mechanisms, including ion-exchange reactions involving peptidoglycan and teichoic acid, nucleation reactions leading to precipitation or the formation of complexes facilitated by ligands. In addition, *Lactobacillus* strains can mitigate the oxidative stress induced by HM toxicity and promote the bio-absorption and detoxification of HMs. The use of probiotics is associated with improvements in intestinal function, resulting in the elimination of HMs and a reduction in their absorption. Moreover, probiotics facilitate detoxification by modulating the expression of proteins involved in metal transport [146]. Table 3 summarises the potential role of a single strain or a combination of different strains of probiotics in HM detoxification. So far, several studies on the efficacy of probiotics have been run on animals. One case in humans was of *Lactobacillus rhamnosus* GR-1 (LGR-1)-supplemented yogurt in pregnant women and children, which protected them from the absorption of As and Hg [147]. In 2022, Feng et al. reported the results of a randomised, double-blind, controlled trial carried out in 152 occupational workers in the metal industry in order to analyse the efficiency of this probiotic yogurt in lowering the levels of HMs. Participants were divided into two different groups, one consuming a probiotic yogurt containing the HM-resistant strain *Pediococcus acidilactici* GR-1, and the other consuming a conventional yogurt for 12 weeks. The consumption of a probiotic yogurt reduced copper (34.45%) and nickel (38.34%) levels when compared to conventional yogurt (16.41% and 27.57%, respectively) [148]. 

Kadry et al. (2018) demonstrated that the administration of *Lactobacillus delbruekii* and *Lactobacillus fermentum* in male Wester mice increased Cd excretion in faeces, increasing the expression of β-catenin and brain-derived neurotrophic factors in the brain tissue and steroidogenic acute regulatory protein and 17-hydroxy steroid dehydrogenase (17-β HSD) in the testes. In addition, supplementation with these probiotics resulted in a high reduction in malondialdehyde and butyrylcholinesterase levels accompanied with an increase in GSH and SOD activities in comparison to the Cd-treated group [149]. Treatment of male Wistar rats with *Lactobacillus plantarum* and *Bacillus coagulans* significantly improved the biochemical parameters, decreasing AST, ALT, total bilirubin, BUN and metal accumulation in the liver and kidneys and increasing body weight, serum and liver SOD values in comparison with the Cd-treated group. When Cd was administered together with a synbiotic diet, there was a significantly lower metal accumulation compared to the Cd-treated group. In the *L. plantarum* + Cd and *B. coagulans* + Cd groups, liver Cd levels were significantly reduced from 23.36 ± 2.27 to 5.44 ± 0.04 and 5.43 ± 0.27 μg/g, respectively. A similar trend was also observed in the Cd levels of the kidneys [150]. Moreover, ten *Lactobacillus strains*, including four *L. plantarum* strains, three *L. fermentum* strains, *L. brevis*, *L. buchneri*, and *L. rhamnosus* were shown to remove Cd from the culture medium [151]. Finally, *L. rhamnosus* Rosell-11, *Lactobacillus acidophilus* Rosell-52 and *Bifidobacterium longum* Rosell-175 strains enhanced Cd concentration in faeces and reduced concentrations in the blood, liver and kidneys by about half, as well as blood ALT and AST activities in comparison to the rat group treated with only Cd. These results suggest that probiotics accelerate Cd elimination through faeces, inducing a reduction in its accumulation in blood and tissues [152]. A different study also confirmed probiotic potential against Pb toxicity. A probiotic strain, *L. plantarum* CCFM8661, markedly induced hepatic bile acid (BA) synthesis, enhanced bile flow and biliary GSH output and inhibited ileal BA re-absorption in male C57BL/6 mice, which, in turn, enhanced Pb faecal excretion. Thus, compared with the control group, a 4-week administration of *L. plantarum* markedly decreased blood Pb levels in mice (about 30% reduction). *L. plantarum* administration also showed a great reduction in hepatic (about 30%) and renal (about 15%) Pb levels in mice than the vehicle treatment [153]. *L. reuteri* P16 alleviated Pb-induced oxidative stress, reversed alterations in haemato-biochemical parameters, improved innate immune parameters and re-established intestinal enzymatic activities in *Cyprinus carpio*. Moreover, Pb exposure markedly increased the level of this metal in gills (5.17 μg/g), spleen (3.86 μg/g), liver (8.92 μg/g), kidneys (26.33 μg/g) and intestines (10.27 μg/g), compared with those in the control group. Supplementation with *L. reuteri* decreased Pb accumulation in all the tissues (about 45% in the gill and spleen, and 35% in the liver and kidneys), except the intestine (only a 13% reduction), as compared with the Pb-treated group. Therefore, *L. reuteri* P16 can be considered for the prevention of Pb contamination aquaculture [154]. Li et al. (2017) demonstrated that *L. bulgaricus* KLDS1.0207 was able to facilitate Pb detoxication in vivo by raising Pb levels in the faeces, improving tissue Pb enrichment, enhancing the antioxidant index and restoring renal toxicity induced by metal exposure. Indeed, in the blood and the kidneys, the decrease in Pb levels for all the dose groups was significant when compared with the values obtained for the Pb group (reduction of 23 and 30%, respectively). Instead, no significant differences were observed in the liver [155]. Finally, another study showed the potential of the probiotic species *Bacillus clausii* against both Pb- and Cd-induced toxicity [156]. Similarly, Daisley et al. (2019) [157] demonstrated the absorbent properties of *Lactobacillus rhamnosus* GR-1 to immobilise Pb and Cd, reducing their translocation in vitro in a Caco-2 model of the intestinal epithelium. Concerning protection against Hg toxicity, a recent study showed how the oral administration of *B. coagulans* and *L. plantarum* restored the levels of GPx and SOD, also reducing the levels of creatinine, urea, bilirubin, ALT and AST. Moreover, both of them decreased Hg levels in the liver and kidneys. In particular, *L. plantarum* and *B. coagulans* caused a 59.16% and 59.8% reduction in levels of Hg in the kidneys, respectively. Concerning Hg levels in the liver, *B. coagulans* and *L. plantarum* induced a decrease of 59 and 52%, respectively, compared to the Hg group [158].

**Table 3:** Heavy metal detoxification with probiotics.

Probiotics	Role in Heavy Metal Detoxification	Ref.
*L. rhamnosus* GR-1	Protection against As and Hg absorption in pregnant women and children	[157]
*P. acidilactici* GR-1	Decrease of Cu (34.45%) and Ni (38.34%) levels	[148]
*L. delbruekii* and *L. fermentum*	Increased Cd excretion when administered with folic acid	[149]
*L. plantarum* and *B. coagulans*	Decreased Cd levels in symbiotic diets along with inulin	[150]
Ten *Lactobacillus strains*, including four *L. plantarum* strains, three *L. fermentum* strains, *L. brevis*, *L. buchneri*, and *L. rhamnosus*	Reduced Cd toxicity	[151]
*L. rhamnosus* Rosell-11, *L. acidophilus* Rosell-52, and *B. longum* Rosell-175 strains	Reduced Cd concentrations in rat blood	[152]
*L. plantarum* CCFM8661	Pb intestinal sequestration by enhancing bile acid production	[153]
*Lactobacillus reuteri* P16	Decreased Pb accumulation in tissues of freshwater fish common carp (*Cyprinus carpio*)	[154]
*Lactobacillus delbrueckii* subsp. *bulgaricus* KLDS1.0207	Increased Pb excretion in mice	[155]
*B. clausii*	Decreased Pb and Cd concentrations	[156]
*L. rhamnosus* GR-1	Pb and Cd sequestration and decreased their absorption across the intestinal epithelium	[157]
*L. plantarum* and *B. coagulans*	Decreased Hg levels in rat livers and kidneys	[158]

Furthermore, several studies reported the ability of probiotics to reduce the availability of HMs in food products [159]. For example, Massoud et al. (2020) [160] discovered that *L. acidophilus* is a naturally efficient biosorbent for Pb and Cd removal from milk in very low concentration levels (parts per billion). *L. acidophilus* was also used for Hg bio-removal from milk and the optimum reduction was 78% after 4 days of refrigeration [161]. Therefore, these probiotic bacteria are potential candidates for protecting the body against foodborne contaminant-induced toxicity [162]. Recently, next-generation probiotics, especially bio-engineered bacteria, have arisen as innovative protective and therapeutic bioagents for the detoxification of HMs [163,164]. In recent years, the application of genetically engineered microorganisms (GEMs) for HM removal has garnered worldwide attention due to their cost-effectiveness, adaptable nature and environmentally friendly characteristics. GEMs with robust degradative capabilities are usually employed for bioremediation purposes, addressing the HMs in groundwater, soil and activated sludge conditions. Typically, the development of effective HM-resistant GEMs is mainly dependant on two different strategies: (1) the creation of surface functional complexes with a strong capacity for HM binding through biosorption, and (2) the transportation of metal ions into the cytoplasm, followed by processing through storage systems to enhance intracellular bio-accumulation. These innovative approaches offer potential avenues for the development of promising biological interventions for HM poisoning [165].

## 5. Conclusions and Perspectives

Silent toxicity induced by HMs in the environment and, consequently, in nutraceuticals and dietary supplements, underlines the importance of quality control, testing and consumer awareness in the dietary supplement industry. Both manufacturers and consumers must prioritise product safety and intervene to minimise the risk of HM contamination in nutraceuticals. It should be recalled that environmental pollution from HMs depends on anthropogenic activities, including industrial and car emissions, chemicals used for agriculture (e.g., Hg in fungicides, lead arsenate in insecticides), battery disposal and so on. In addition, numerous variables such as geoclimatic conditions, soil physicochemical characteristics and exposure periods, strongly influence the HM levels in nutraceutical/food sources. Phytochemicals are natural compounds found in plants, showing various biological activities, including antioxidant and detoxifying properties. Certain phytochemicals may help to mitigate or counteract the harmful effects of HMs on biological systems. These phytochemicals can chelate HMs, reducing their bioavailability and toxicity. Several phytochemicals may have antioxidant properties, which can protect cells and tissues from oxidative damage induced by HMs. Hence, studying the interactions between phytochemicals and HMs is very important for understanding potential strategies to prevent or alleviate HM toxicity in various contexts, such as environmental exposure or nutrition and nutraceuticals. On the basis of the studies summarised in this review, a daily intake of nutraceuticals is advisable, mostly for people chronically exposed to HMs, in order to diminish the risk of disease onset because of HM toxicity. Indeed, dietary supplements represent an ideal option with respect to chelation therapy, which is not devoid of important side effects. Moreover, since the contamination of the food chain may present an indirect source of HM toxicity for humans, urgent interventions from the health authorities are needed to avoid the spread of this phenomenon. Several studies describe the beneficial effects of different nutraceuticals in human trials; however, the lack of sufficient information on the adopted doses, bioavailability, pharmacokinetics and pharmacodynamics or the potential risk of adverse effects due to a hyper-dosage is still a stumbling block to overcome. Finally, long-term epidemiological studies aimed at determining the optimal dose of nutraceuticals or supplements, alone or in combination, are needed to pave the way toward an effective dietary strategy to face HM toxicity.

## Figures and Tables

**Table 2:** Protective effects of *Spirulina* against heavy metal-induced toxicity.

Pollutant	Test Spice	*Spirulina* Treatment	Effect	Ref.
Arsenic	Human	250 mg plus 2 mg zinc twice daily in drinking water (16 weeks)	Removal of 47.1% As from scalp hair with melanosis and keratosis improvement	[119]
Arsenic	Human	10 mg daily dissolved in water (6 months)	Reversal of health conditions and restored the patients to normal life	[121]
Arsenic	Duck	30–120 mg/L in drinking water (90 days)	Enhanced body weight and restored haematological parameters	[122]
Cadmium	Mice	62.5–500 mg/kg, p.o. (gestation days 0–17)	Decreased frequency of exencephaly and other foetus malformations	[123]
Cadmium	Rat	500 mg/kg/d p.o. (30 days)	Partially prevented lowering of metal serum concentrations (zinc, iron and selenium). Protective capacity against liver and renal damage due to the antioxidant activity	[124]
Cadmium	Rat	300 mg/kg p.o. (30 days)	Protection against liver damage due to its ability to reduce the vacuolar degeneration, fat infiltration and fibrosis	[125]
Lead	Mouse	800 mg/kg p.o. (15 days before and up to 30 after intoxication)	Decreased affectation on animal and testes weights and tubular diameter, improving the survival time	[126]
Lead	Rat	1500 mg/kg in diet (30 d)	Enhanced SOD, CAT and GSH in liver, lungs, heart and kidneys; in addition, reduced brain metal concentrations and LPO	[127]
Lead	Rat	300 mg/kg in drinking water (30 days)	Reduced increase in the number of mast cells in the ovarian cortex and medulla during the oestrous cycle	[128]
Lead	Rat	20 g diet 5%/d/rat (30 days)	Prevented body weight reduction and liver impairment and protection against oxidative damage in liver and kidneys	[129]
Lead	Rat	5% + dandelion 2% in diet (5th day of gestation to 14th day of lactation)	Minimised lead deposition and oxidative stress in gestation and lactation	[130]
Mercury	Mouse	800 mg/kg p.o. (before and after HgCl_2_ exposition)	Reduced activity of ACP and ALP in testicles	[131]
Mercury	Mouse	800 mg/kg p.o. (10 days before and 30 days after intoxication)	Modulation of biochemical alterations in blood: calcium and ion levels, acid and alkaline phosphatase activity and lipid peroxidation and GSH level	[132]
Mercury	Mouse	800 mg/kg p.o. (10 days before and 30 days after intoxication)	Protection against renal damage reducing LPO, acid phosphatase activity, tissue degeneration and increased ALP, lactate dehydrogenase and GSH levels	[133]
Mercury	Rat	300 mg/kg p.o. (10 days before and 60 days after)	Reduced hepatotoxicity as well as altered lipid profile through its antioxidant activity	[134]
Mercury	Rat	300 mg/kg p.o. (10 days before and 60 days after)	Protection against testicular damage, re-establishing oxidative stress biomarkers, sperm quality and histopathological alterations	[135]

## Data Availability

No new data were created or analyzed in this study. Data sharing is not applicable to this article.
